# Chiral polymer modified nanoparticles selectively induce autophagy of cancer cells for tumor ablation

**DOI:** 10.1186/s12951-018-0383-9

**Published:** 2018-07-11

**Authors:** Long Yuan, Fan Zhang, Xiaowei Qi, Yongjun Yang, Chang Yan, Jun Jiang, Jun Deng

**Affiliations:** 1 Department of Breast Surgery, Southwest Hospital, Third Military Medical University (Army Medical University), Chongqing, 400038 China; 2 Medical Research Center, Southwest Hospital, Third Military Medical University (Army Medical University), Chongqing, 400038 China; 3 Department of Cardiology, Southwest Hospital, Third Military Medical University (Army Medical University), Chongqing, 400038 China; 4 Institute of Burn Research, Southwest Hospital, State Key Lab of Trauma, Burn and Combined Injury, Third Military Medical University (Army Medical University), Chongqing, 400038 China

**Keywords:** Nanomaterials, Chiral polymer, Cytotoxicity, Autophagy, Tumor ablation

## Abstract

**Background:**

Autophagy regulation through exogenous materials has aroused intensive attention to develop treatment protocols according to diverse human diseases. However, to the best of our knowledge, few examples have been reported to selectively control autophagy process and ultimately achieve efficient therapeutic potential.

**Results:**

In this study, monolayers of poly (acryloyl-l, d and racemic valine) (l-PAV-AuNPs, d-PAV-AuNPs and l/d-PAV-AuNPs) chiral molecules were anchored on the surfaces of gold nanoparticles (PAV-AuNPs), and the subsequent chirality-selective effects on autophagy activation were thoroughly studied. The cytotoxicity induced by PAV-AuNPs towards MDA-MB-231 cells (Breast cancer cells) was achieved mainly through autophagy and showed chirality-dependent, with d-PAV-AuNPs exhibiting high autophagy-inducing activity in vitro and in vivo. In contrast, the PAV-AuNPs exhibited autophagy inactivation for normal cells, e.g., 3T3 fibroblasts and HBL-100 cells. The chirality-selective autophagy activation effect in MDA-MB-231 cells was likely attributed to the chirality-variant ROS generation, cellular uptake and their continuous autophagy stimulus. Furthermore, the intratumoral injection of d-PAV-AuNPs could largely suppress the tumor growth but exhibit negligible toxicity in vivo.

**Conclusions:**

As the first exploration on stereospecific NPs for autophagy induction, this work not only substantiates that chiral polymer coated NPs can selective induce autophagy-specific in cancer cells and achieve a high tumor eradication efficiency in vivo, but also opens up a new direction in discovering unprecedented stereospecific nanoagents for autophagy-associated tumor treatment.

**Electronic supplementary material:**

The online version of this article (10.1186/s12951-018-0383-9) contains supplementary material, which is available to authorized users.

## Background

Breast cancer is the most frequent carcinoma in females and the second common cause of cancer-related mortality in women. Moreover, about 63,410 cases of carcinoma in situ of the female breast will be newly diagnosed in the United States in 2017 [1]. Particularly, approximately 10–20% of breast cancer cells are triple-negative breast cancer (TNBC), which do not express estrogen receptor, epidermal growth factor receptor 2 and progesterone receptor [2]. Until now, TNBC is regarded as an aggressive type of breast cancer with drug resistance, high metastatic and tumor relapse rates [3–5]. The treatment for TNBC is extremely difficult due to the lack of validated molecular targets, prognostic and therapeutic markers. Even more worse, the current treatment for the TNBC including chemotherapy [6] and radiation therapy [7] are severe short and long term side effects due to the short of efficacious selectivity of differentiating between cancerous and normal cells. Therefore, there is an urgent need for development of alternative effective therapies for treatment of TNBC to overcome the drawbacks above.

Autophagy is a catabolic process, and plays critical roles for cytoplasmic quality control including maintaining cellular homeostasis and contributing to cellular defense [8]. Generally, through autophagy, endogenous as well as foreign materials are sequestered in vesicles (e.g., autophagosomes), and finally degraded upon fusion of these autophagic vesicles with lysosomes [9]. Recently, scientists have attempted to manipulate autophagy and inhibit further tumor progress. Compared with present cancer therapy strategies including chemo-, immuno-, gene, photothermal, photodynamic and radiation therapies, the autophagy-based conceptual pilot studies for cancer therapy are of particular-and growing-interest in autophagy-deficient cancers, such as breast, ovarian, and prostate cancers. Therefore, effective control of the autophagy is expected to be used for treatment of TNBC.

The advancement of nanotechnology allows the fabrication of materials at the nanoscale, which provides these materials with the potential ability to modulate/influence cellular systems in unforeseen ways [10–12]. Compared with the traditional drugs such as growth factors and proteins/peptides, nanomaterials possess some unique properties including excellent photo, thermal and magnetic performance, and advantages including easy to synthesize, lower risk of drug resistance and cheaper [13–15]. Recently, nanoparticles (NPs), considered as foreign materials for cells, have been reported that could influence autophagy process [9]. However, to the best of our knowledge, few examples have been reported to effectively control autophagy process and ultimately achieve therapeutic potential. Thereby, alternative NPs or surface modifications are urgently required to achieve the above objectives.

Chirality is an important and common phenomenon in living systems. For instance, almost all of amino acids (except glycine) in proteins are “left-handed” (l-), while all sugars in DNA and RNA are “right-handed” (d-) [16]. Our and other previous studies have demonstrated chirality-dependent nature of cellular uptake [17], cell adhesion [18], cell differentiation [19, 20], protein adsorption [21, 22] (i.e., amount and affinity) and cytotoxicity [23] on flat substrates or nanoparticles coated with chiral molecules. More recently, two studies observed that NPs with surface-anchored chiral molecules could modulate the autophagy [24, 25]. However, the conclusions, interestingly, are diametrically opposed, which was possibly due to the observed weak chiral effects on autophagy. To employ the chirality-dependent activation of autophagy in cancer treatment, there are two important issues need to be understand. Firstly, the efficiency of regulation of autophagy for tumor eradication especially in vivo needs to be largely improved. Secondly, the NP coated with chiral molecules should possess the ability to selectively induce autophagy between normal cells and cancer cells.

Herein, to address these challenges, we describe the development of autophagy-inducing gold nanoparticles (AuNPs) coated with chiral poly (acryloyl-l, d and racemic valine) (l-PAV-AuNPs, d-PAV-AuNPs and l/d-PAV-AuNPs) for highly efficient chiral selectivity induction of autophagy in MDA-MB-231 cells (triple-negative breast cancer cells) and eradication of TNBC in vitro and in vivo. The chirality-selective autophagy activation effect in MDA-MB-231 cells was likely attributed to the chirality-variant ROS generation, cellular uptake and their continuous autophagy stimulus. In contrast, the PAV-AuNPs exhibit autophagy inactivation in two model normal cells, 3T3 fibroblasts and HBL-100 cells (normal breast epithelial cells), regardless of surface chirality. Moreover, the PAV-AuNPs possess excellent biocompatibility in vitro and in vivo. To the best of our knowledge, we for the first time reported that simultaneously employing of the chirality-dependent and chirality-selectivity activation of autophagy could be used in tumor ablation.

## Experimental section

### Materials

Gold(III) chloride hydrate (HAuCl_4_) and sodium citrate (C_6_H_5_Na_3_O_7_·2H_2_O) were purchased from Sinopharm group Co. Ld. Poly(acryloyl-l-valine) (l-PAV) (*M*_w_ 4926 Da, polydispersity 1.24), poly(acryloyl-d-valine) (d-PAV) (*M*_w_ 4997 Da, polydispersity 1.15) were synthesized and were characterized according our previous works [17, 23]. High glucose dulbecco’s modified eagle medium (DMEM) and fetal bovine serum (FBS) were obtained from HyClone (USA). Cell counting Kit-8 (CCK8) assay was purchased from DOJINDO Company (Japan). Acridine Orange (AO), 3-methyladenine (3-MA) and primary antibody (Anti-LC3B) (antibody produced in rabbit, L7543) were obtained from Sigma-Aldrich (USA). Annexin V-FITC/PI Kit was bought from BestBio (Shanghai, China). Cyto-ID Green Kit was purchased from ENZO (USA). 2,7-Dichlorodi-hydrofluorescein diacetate (DCFH-DA), radio immunoprecipitation assay (RIPA) lysis buffer penicillin–streptomycin and secondary antibody (Anti-Rabbit IgG (H + L), A0208) were obtained from Beyotime (China). The Milli-Q water was used throughout the experiments.

### Synthesis and characterization of L(D)-PAV-AuNPs

The synthesis of poly(acryloyl-l, d and racemic valine) coated gold nanoparticles (l-PAV-AuNPs, d-PAV-AuNPs, l/d-PAV-AuNPs) with diverse surface chirality can be found in previous studies [17, 23]. Briefly, for the modification of AuNPs with PAV, AuNPs were mixed with excess l-PAV or d-PAV or a mixture of l-PAV and d-PAV with equal quantities. The stock solutions of PAV-AuNPs were obtained by centrifugation at 9000 rpm for 5 min to remove nondispersive aggregates from the suspensions, and dialyzed with odium phosphate solution. The mass concentration (μg/L) of l(d)-PAV-AuNP solution was determined using inductively coupled plasma mass spectrometry (ICP-MS, XSENIES, USA). The nanoparticle (NP) geometry was characterized by transmission electron microscopy (TEM, JEM-1400PLUS). The hydrodynamic diameters of PAV-AuNPs were obtained using dynamic light scattering (DLS) with a high performance particle size analyzer (Zetasizer Nano, Malvern) at room temperature (25 °C) with a fixed detector angle of 173°. The UV–Vis and circular dichroism (CD) spectra were measured with a UV–Vis-NIR spectrophotometer (Shimadzu UV-3600) and a Jasco J-810 spectropolarimeter at room temperature according our previous works [17], respectively.

### In vitro experiments

MDA-MB-231 cells, 3T3 fibroblasts and HBL-100 cells were obtained from Cell Bank of Chinese Academy of Sciences, Shanghai Branch (Shanghai, China), and routinely cultured in DMEM containing 10% of FBS and 1% penicillin–streptomycin. Cells were cultured at 37 °C with environment of 95% air and 5% CO_2_.

#### Cell viability assay

Cell viability was determined using a CCK-8 assay according to the manufacturer’s specification. Briefly, MDA-MB-231 cells, 3T3 fibroblasts and HBL-100 cells were seeded on a 48-well plate at a density of 2 × 10^4^ cells/cm^2^ and allowed to attach for 24 h. Then, the culture medium was replaced with 10% FBS/DMEM containing PAV-AuNPs with various Au concentration (5–200 μg/mL), respectively. After being co-cultured for another 24 h, the plates were washed 3 times with phosphate buffered saline (PBS). The CCK-8 reagent was added to each well and incubated for another 2 h, and then the absorbance at 450 nm was measured using a microplate reader (Varioskan Flash, Thermo Scientific, USA).

#### Cellular uptake of PAV-AuNPs

The amount of PAV-AuNPs internalized by MDA-MB-231 cells was determined by ICP-MS according to our previous reported protocol [17]. The Au amount per 10^4^ cells from ICP-MS analysis was presented as the mean ± standard deviation.

#### Apoptosis assay

For Annexin V-FITC/propidium iodide (PI) assays, cells were stained and analyzed using the flow cytometry (FCM, NovoCyte, ACEA Biosciences, USA) in line with the manufacturer’s instruction [26].

#### Autophagy assay

The cells were seeded on a 6-well plate at a density of 2 × 10^5^ cells/cm^2^ to attach for 24 h. On the one hand, the cells were pre-treated with or without 3-MA (5 mmol/L) for 1 h before changing the medium containing PAV-AuNPs with an Au concentration of 100 μg/mL. After being co-cultured with PAV-AuNPs for 24 h, the cell viability was determined using CCK-8 assay. On the other hand, after being incubated with PAV-AuNPs for 24 h, cells were stained using Cyto-ID Green Kit according to the manufacturer’s instruction. Then, the cells were imaged using fluorescence microscope (DM 6000 B, Leica, Germany). For quantitatively study, cells were tested using FCM to assess the level of autophagy.

#### Reactive oxygen species (ROS) generation

The intracellular generation of ROS was measured with a procedure similar to the apoptosis assay. After being treated with PAV -AuNPs (100 μg/mL), then cells were washed with PBS for three times and loaded with 1 mL of 10 μM DCFH-DA for further 20 min incubation. Next, cells were washed for three times with DMEM without FBS to get rid of the DCFH-DA outside cell membrane. Finally, the cells were measured using FCM (excitation at 488 nm and emission at 525 nm).

#### Lysosomal membranes assay

To examine the release of AO, from lysosomes into the cytosol, the procedure was performed identically to the protocol of ROS generation. Briefly, after being treated with PAV-AuNPs for 24 h, cells were then incubated with 5 µg/mL of Acridine Orange (AO, Sigma) for additional 15 min at 37 °C. After being washed with PBS three times, the cells were then harvested by trypsinization and measured using FCM (excitation at 488 nm and emission at 525 nm).

#### Western blotting analysis

MDA-MB-231 cells, 3T3 fibroblasts and HBL-100 cells were seeded in 6-well plates at a density of 1 × 10^6^ cells/mL in the presence of PAV-AuNPs with an Au concentration of 100 μg/mL at 37 °C for 24 h. Following, the cells were treated as described [27]. In the present study, anti-LC3B (1:1000, Sigma, USA) was used for primary antibodies, subsequently with an appropriate secondary antibody (1:5000, Beyotime, China). Glyceraldehyde-3-phosphate dehydrogenase (GAPDH) was used as the internal control.

### Animals experiments

Animal care and use were conducted according to the Third Military Medical University (Army Medical University) for the Care and Use of Laboratory Animals. BALB/C mice and nude mice (6–8 weeks old), ranging from 20 to 25 g, were purchased from BEIJING HFK BIOSCIENCE CO.LTD.

#### In vivo treatment study

After 7 days, mice were randomly assigned to 3 groups (5 animals per group) upon tumors reached a diameter of 4 to 6 mm [28] and intratumorally injected with 50 μL of 1 mg/mL l(d)-PAV-AuNPs every other day. For control groups, mice were treated with the same volume of physiological saline. The tumor sizes were measured by a caliper every day and calculated as the volume (*V*, tumor length × (tumor width)^2^/2). The data was presented as relative tumor volume which was calculated as *V*/*V*_0_ (*V*_0_ is the tumor volume before the treatment).

#### Biodistribution exploration

The in vivo biodistribution of NPs was analyzed by testing the Au content in main organs (liver, kidneys, spleen, heart, and lung). The mice were injected with 0.5 mL of L(D)-PAV-AuNPs (1 mg mL) via the tail vein. After being treated for a certain time (1 or 30 days), organs were collected and washed with PBS buffer and lyophilized for 1 day. Subsequently, the dried tissues were pulverized and dissolved in aqua regia (HCl/HNO_3_ = 1:3, volume ratio) for 7 days. Then, the Au content was analyzed using ICP-MS.

#### Transmission electron microscopy (TEM) analysis

After being treated with 100 µg/mL of l(d)-PAV-AuNPs for 24 h, cells were collected, centrifuged, and then fixed with 2.5% (*v/v*) glutaraldehyde. After fixation for 2 h at 4 °C, the samples were washed with PBS three times. Then, the samples were fixed with 1% perosmic oxide, and dehydrated in an alcohol series, embedded, and sliced with the thickness between 50 and 70 nm. Finally, the images were obtained by using the TEM analysis (JEM-1400PLUS).

#### In vivo toxicity assay

After being injected with 0.5 mL of l(d)-PAV-AuNPs, the mice were sacrificed at 1 or 30 days. Afterwards, main organs (liver, kidneys, spleen, heart, and lung) were gathered, washed with PBS, and immobilized in 4% paraformaldehyde overnight. Then, the samples were embedded with paraffin, sliced into 4 μm, and stained with hematoxylin and eosin (H&E). Simultaneously, blood samples were subjected for the measurement of hematology and biochemistry. The organ (Spleen) index defined in our research as the weight percentage of the spleen to the body was calculated.

### Statistical analysis

The experimental data are expressed as mean ± standard deviation, and the significant difference between groups was analyzed by using one-way analysis of variance (ANOVA) (for two groups) and two-way ANOVA (for more than two groups) in the Origin software. The statistical significance was set as *p *< 0.05 and p < 0.01, respectively.

## Results

**Fig. 1:**
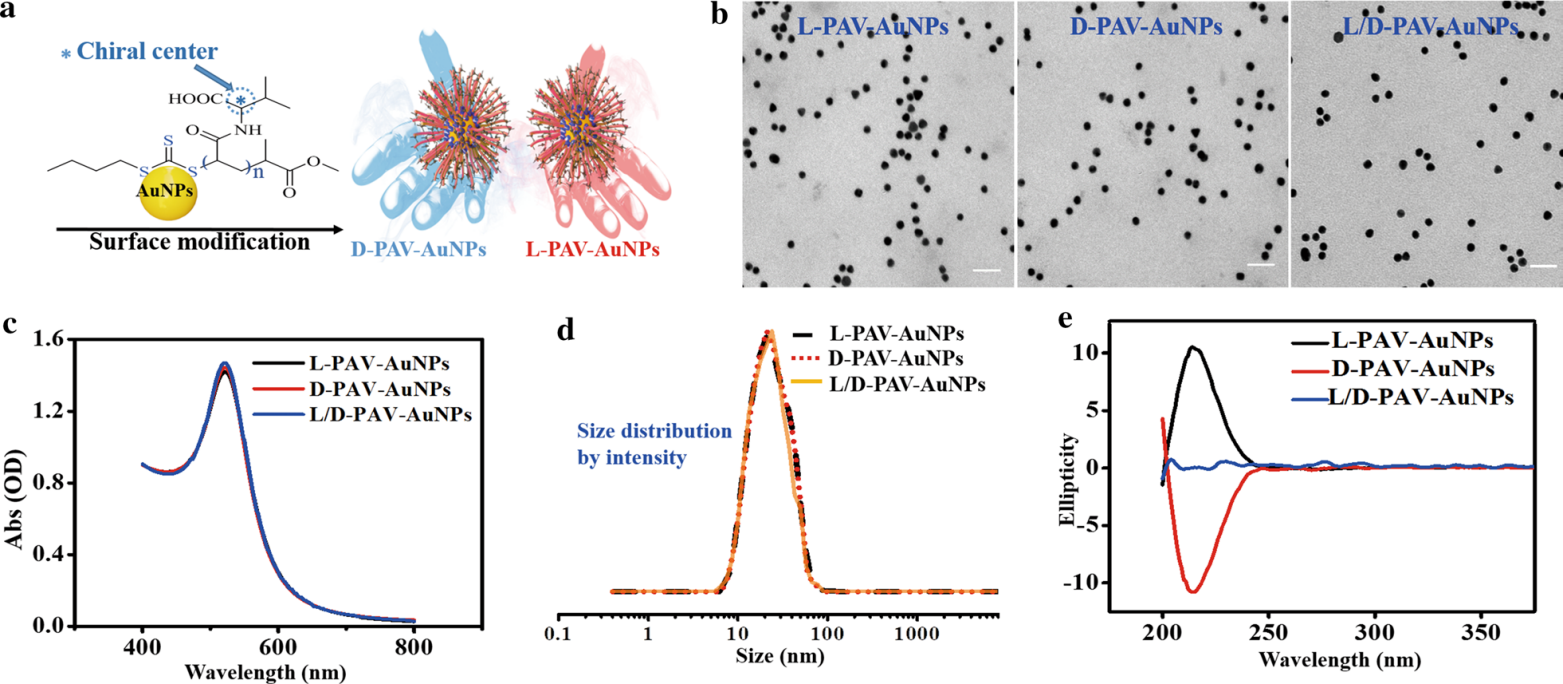
Characterizations of l-PAV-AuNPs, d-PAV-AuNPs l/d-PAV-AuNPs. **a** Schematic illustration of the synthesis of poly (acryloyl-l, d and racemic valine) (l-PAV-AuNPs, d-PAV-AuNPs l/d-PAV-AuNPs) and their conjugation to the AuNP surface. **b** Preventative TEM images of PAV-AuNPs. **c** UV–Vis-NIR spectra of PAV-AuNPs in PBS. **d** The curves of the size distribution by in intensity of PAV-AuNPs. **e** Circular dichroism (CD) spectra of PAV-AuNPs

In this study, chiral PAV molecules, composed of valine (one of the eight essential amino acids of human body), were grafted on the AuNPs through the thioester-Au bond [17] (Fig. 1a). Although the more chiral centers exist in PAV molecules, the chiral center of monomer repeating units accounts for the vast majority (~ 97%). Moreover, in general, the chiral centers generated during polymerization are racemization [29–31]. However, the main chain may also form a spiral conformation due to the induction of the side chain groups, which makes the chiral carbons of the main chain produced in the process of the polymerization show certain chirality [29, 30]. Steric hindrance from the side groups is beneficial to the formation and stability of helical conformation of polymer backbone [30]. While the side group valine in poly(acryloyl-l(d)-valine) presents weak steric hindrance [32, 33], which is not easy to form and stabilize the helical conformation of the main chain of polymer. Therefore, we believe that the main configuration of these chiral centers should be the chiral center of monomer repeating unit of the side valine group, which was marked with asterisk in Fig. 1a and discussed in text. Characterization by transmission electron microscope (TEM) analysis showed that PAV-AuNPs were approximately spherical in shape and had an average-diameter of 15 nm (Fig. 1b), regardless of surface chirality. The UV–Vis-NIR spectra exhibited that PAV-AuNPs had a surface plasmon resonance (SPR) peak of 521 nm (Fig. 1c). The DLS results showed that l-PAV-AuNPs, d-PAV-AuNPs and l/d-PAV-AuNPs had a similar size of 20 nm (Fig. 1d). The zeta potential consequence suggested that L-PAV-AuNPs, d-PAV-AuNPs and l/d-PAV-AuNPs had a similar negative surface charge in water (~ − 29 mV) (Table 1). l-PAV-AuNPs and d-PAV-AuNPs showed essentially mirror-image circular dichroism (CD) spectra; while the negative CD spectra was observed for l/d-PAV-AuNPs in the region of 200–300 nm, which indicated that the l/d-PAV-AuNPs to be the racemic specialty (Fig. 1e). Moreover, PAV-AuNPs were found to be excellent stable and no significant degree of aggregation in various physiological solutions including saline, PBS, cell culture medium containing 10% fetal bovine serum (10% FBS/DMEM), FBS and dilution of whole blood of mice (whole blood of the mice: heparin dilution liquid = 1:8) for 3 days (Additional file 1: Figure S1). The UV–Vis-NIR spectra of PAV-AuNPs in various physiological solutions had a parallel surface plasmon resonance (SPR) peak (Additional file 1: Figure S1), which further demonstrated the excellent stability of the PAV-AuNPs in above solutions. Taking into account of these results, it can be concluded that the only difference between these two types of NPs is their surface chirality.

**Table 1:** Characterization of chiral PAV-modified gold nanoparticles

NPs	Diameter (TEM, nm)	Diameter (DLS, Z-average, nm)		Zeta potential (mV)	
		PBS	10% FBS/DMEM	PBS	10% FBS/DMEM
l-PAV-AuNPs	15.1 ± 0.6	20.2 ± 1.7	23.7 ± 2.3	− 29.1 ± 1.9	− 8.8 ± 3.6
d-PAV-AuNPs	15.2 ± 0.2	20.3 ± 1.4	23.8 ± 3.6	− 29.2 ± 3.2	− 8.9 ± 3.2
l/d-PAV-AuNPs	15.1 ± 0.7	20.3 ± 0.8	23.8 ± 1,1	− 29.1 ± 2.4	− 8.9 ± 2.2

**Fig. 2:**
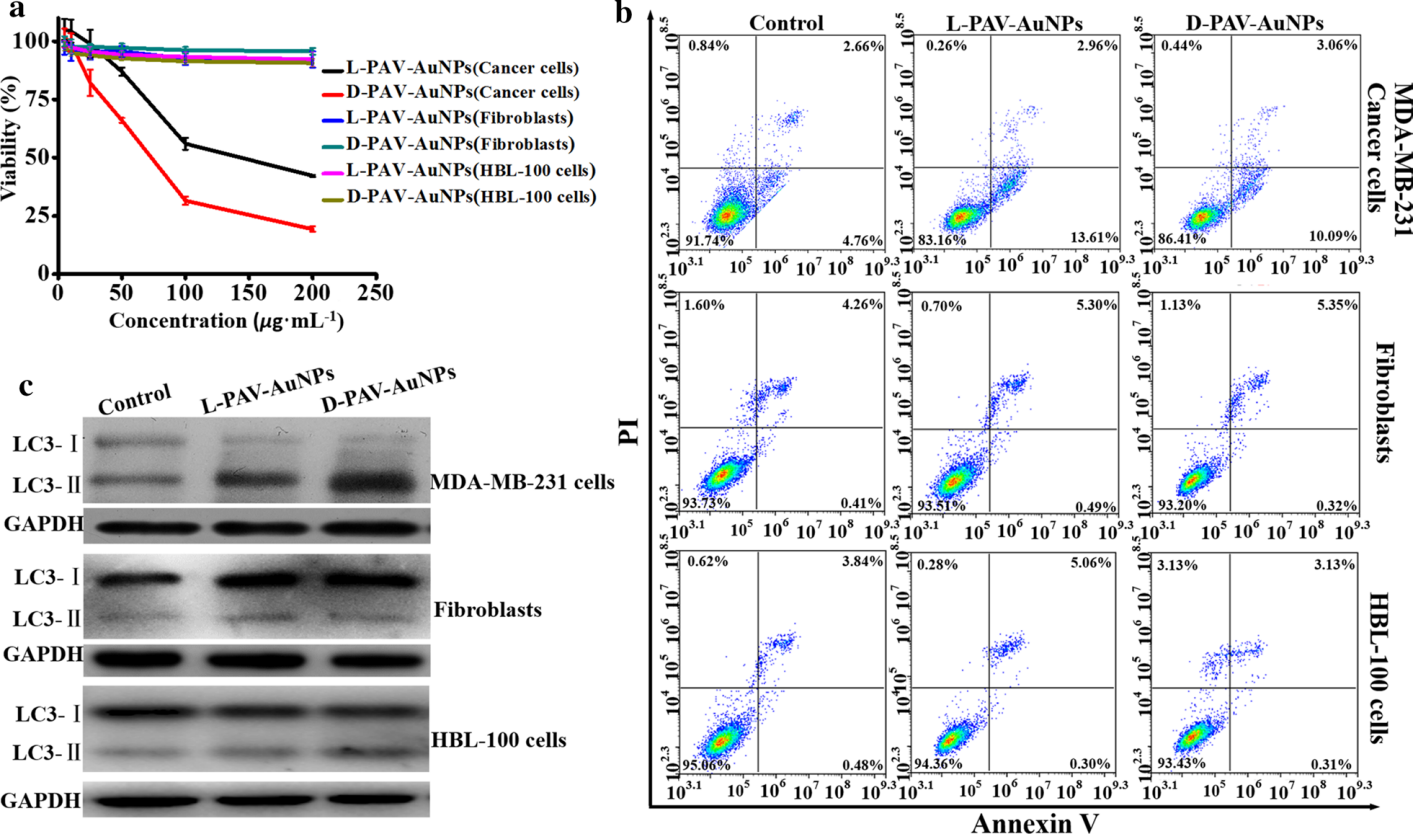
The toxicity study of l-PAV-AuNPs, d-PAV-AuNPs. **a** Dose- and chirality-dependent cytotoxicity of PAV-AuNPs in MDA-MB-231 cells, 3T3 fibroblasts and HBL-100 cells, respectively. **b** Apoptosis rates of the MDA-MB-231 cells, 3T3 fibroblasts and HBL-100 cells treated with PAV-AuNPs. FCM analysis was tested via Annexin V-FITC and PI as probes. **c** Expression levels of LC3 in MDA-MB-231 cells, 3T3 fibroblasts and HBL-100 cells with PAV-AuNPs treatment. GAPDH was used as a loading control

To explore the different biochemical effects of l-PAV-AuNPs, d-PAV-AuNPs and l/d-PAV-AuNPs, we first compared their effects on cell viability in MDA-MB-231 cells, 3T3 fibroblasts and HBL-100 cells. As shown in Fig. 2a, PAV-AuNPs showed concentration- and chirality-dependent cytotoxicity, with d-PAV-AuNPs showing greater cytotoxicity and significant inhibitory potency (IC_50_, l-PAV-AuNPs: 143.56 µg/mL, d-PAV-AuNPs: 71.90 µg/mL, l/d-PAV-AuNPs: 94.26 µg/mL) for a given concentration in MDA-MB-231 cells. On the contrary, there was no obvious cytotoxicity and inhibitory potency (IC_50_, PAV-AuNPs: > 1000 µg/mL) in 3T3 fibroblasts and HBL-100 cells treated by PAV-AuNPs even at a high concentration of 200 µg/mL.

These caused us keen interest how the chirality elicited the nanotoxicity leading to MDA-MB-231 cells death. As we all know, the process of cell death is very complex, and during its progress, damaged cells typically present various markers representative of different cell death pathways, mainly including apoptosis and autophagy [9]. Apoptosis is an intrinsic self-killing process that is necessary for maintaining tissue homeostasis. Autophagy is a cytoprotective mechanism that responds to various stresses conditions such as starvation, radiation, hypoxia and chemotherapeutic drugs to adapt to environmental changes [34]. In several studies, NPs have been found to be capable of inducing cellular responses of autophagy and apoptosis [35–37], so we further systematically explore the underlying mechanism of PAV-AuNPs-induced nanotoxicity.

Apoptosis is commonly identified as programmed cell death-1, while an autophagic cell death is generally deemed as programmed cell death-2 [38]. Thus, apoptosis of PAV-AuNPs treated cells was measured firstly by flow cytometry (FCM). As shown in Fig. 2b and Additional file 1: Figure S2, relative to the untreated control group, only few apoptotic cells (early and late apoptosis) were detected in MDA-MB-231 cells, 3T3 fibroblasts or HBL-100 cells with PAV-AuNPs treatment, and the activation of apoptosis was not chirality-independent. In short, it was deduced that apoptosis might not be the principal reason giving rise to the nanotoxicity of PAV-AuNPs in MDA-MB-231 cancer cells. Analogously, Wu et al. reported that cytotoxicity induced by NPs was through autophagy rather than apoptosis [39]. Therefore, we speculate that the autophagy may play a major role in the process of PAV-AuNPs-induced nanotoxicity in MDA-MB-231 cancer cells.

**Fig. 3:**
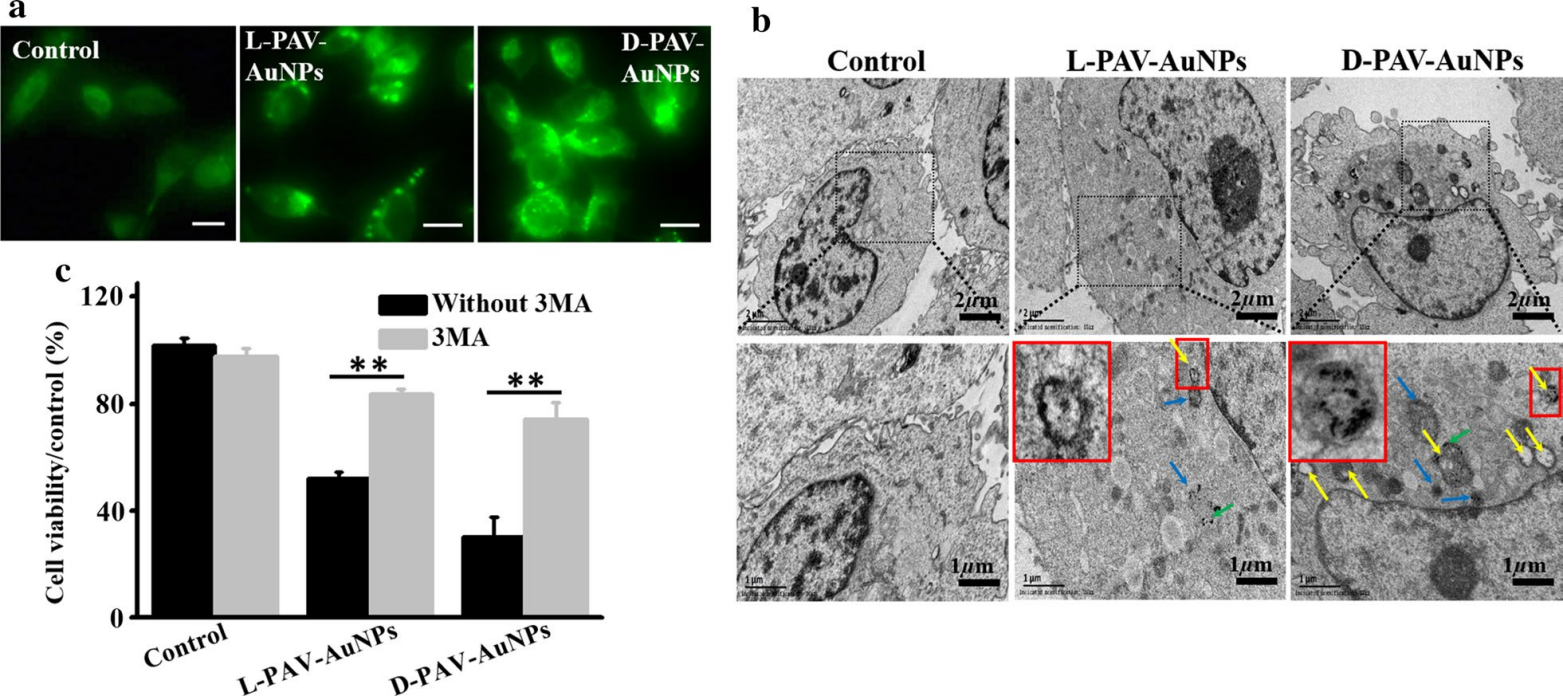
Activation of autophagy induced by l-PAV-AuNPs and d-PAV-AuNPs, respectively. **a** Cyto-ID Green dye labeled dots formed in MDA-MB-231 cells incubated with l- and d-PAV-AuNPs. Scale bar is 50 µm. **b** TEM images of the MDA-MB-231 cells incubated with PAV-AuNPs. The untreated cells were considered as control. The PAV-AuNPs form distinct, dark aggregates in the cells (green arrows). The PAV-AuNPs were localized in lysosomes (blue arrows) and autophagosomes (yellow long arrows), consisting of double layered membranes containing cellular debris. **c** The viability of MDA-MB-231 cells was measured after the cells treated with pre-treatment with or without 3-MA (5 mM) for 1 h. * and ** present p < 0.05 and p < 0.01, respectively

To verify our hypothesis above, autophagic markers of which the most widely examined were deeply detected. For instance, LC3, which is selectively incorporated into autophagic membranes, has been the most specific and therefore also the most analyzed autophagy marker [9]. In comparison with untreated controls, PAV-AuNPs triggered conversion of microtubule-associated protein light chain 3 (LC3)-I to LC3-II in MDA-MB-231 cells (Fig. 2c and Additional file 1: Figure S2), the best-characterized marker of autophagy [40, 41]. This activation of autophagy was chirality-variant, with d-PAV-AuNPs inducing more dramatic autophagy than l-PAV-AuNPs and l/d-PAV-AuNPs, and l/d-PAV-AuNPs inducing slightly higher autophagy than l-PAV-AuNPs (Fig. 2c and Additional file 1: Figure S2). Interestingly, the activation of autophagy induced by PAV-AuNPs in 3T3 fibroblasts and HBL-100 cells still were not observed (Fig. 2c and Additional file 1: Figure S2), irrespective of the surface chirality. The consequence above demonstrated that PAV-AuNPs exhibit selective autophagy activation between cancer cells (MDA-MB-231) and normal cells (3T3 fibroblasts and HBL-100 cells), which can be used as a novel therapeutic strategy for tumors ablation. We speculate that the selective activation of autophagy induced by the PAV-AuNPs between cancer cells and normal cells was possibly attributed to the differences of biomolecules or proteins in their membranes, which may influence their interactions with the NPs (i.e., cellular uptake). Subsequently, we further deeply explore the activation of autophagy induced by the PAV-AuNPs in cancer cells. Furthermore, autophagosomes can be selectively detected by distinct puncta of GFP-LC3 transfection in MDA-MB-231 cells, an increased number of punctate structures per cell thus correlates with autophagy induction [40]. To further confirm induction of autophagy being a chirality-dependent process, we selected l-PAV-AuNPs and d-PAV-AuNPs inducing distinct effects of autophagy to further explore the effects of PAV-AuNPs on MDA-MB-231 cells stably overexpressing LC3-EGFP (EGFP: enhanced green fluorescent protein). Fluorescence microscopy showed that both l-PAV-AuNPs and d-PAV-AuNPs induced LC3-EGFP aggregation in MDA-MB-231 cells, with the effect was stronger for d-PAV-AuNPs (Fig. 3a). To more precisely define the morphological changes and cellular vacuole formation accompanying NP treatment, MDA-MB-231 cells treated with PAV-AuNPs for 24 h were examined by TEM, which allows visual detection of the process based on the specific morphology of the autophagic organelles (double-membraned autophagosomes and autophagosomal cargo). The morphological observations on the formation of large numbers of autophagic vacuoles (Fig. 3b) are consistent with increases in conversion of LC3-I to LC3-II (Fig. 2c) and LC3-EGFP fluorescence intensity (Fig. 3a). As shown in Fig. 3b (yellow arrows), numerous large vacuoles were observed in the cytoplasm of NP-treated cancer cells, which were the features of autophagic cell death. More importantly, d-PAV-AuNPs were more effectively at accumulating vacuole formation than l-PAV-AuNPs. Compared to previously observed weak chiral effects on autophagy, the high efficiency of regulation of autophagy in cancer cells was probably due to the chiral polymer coating, which could enhance the chiral effect on biological behaviors (e.g., cellular uptake, nanotoxicity) [42].

**Fig. 4:**
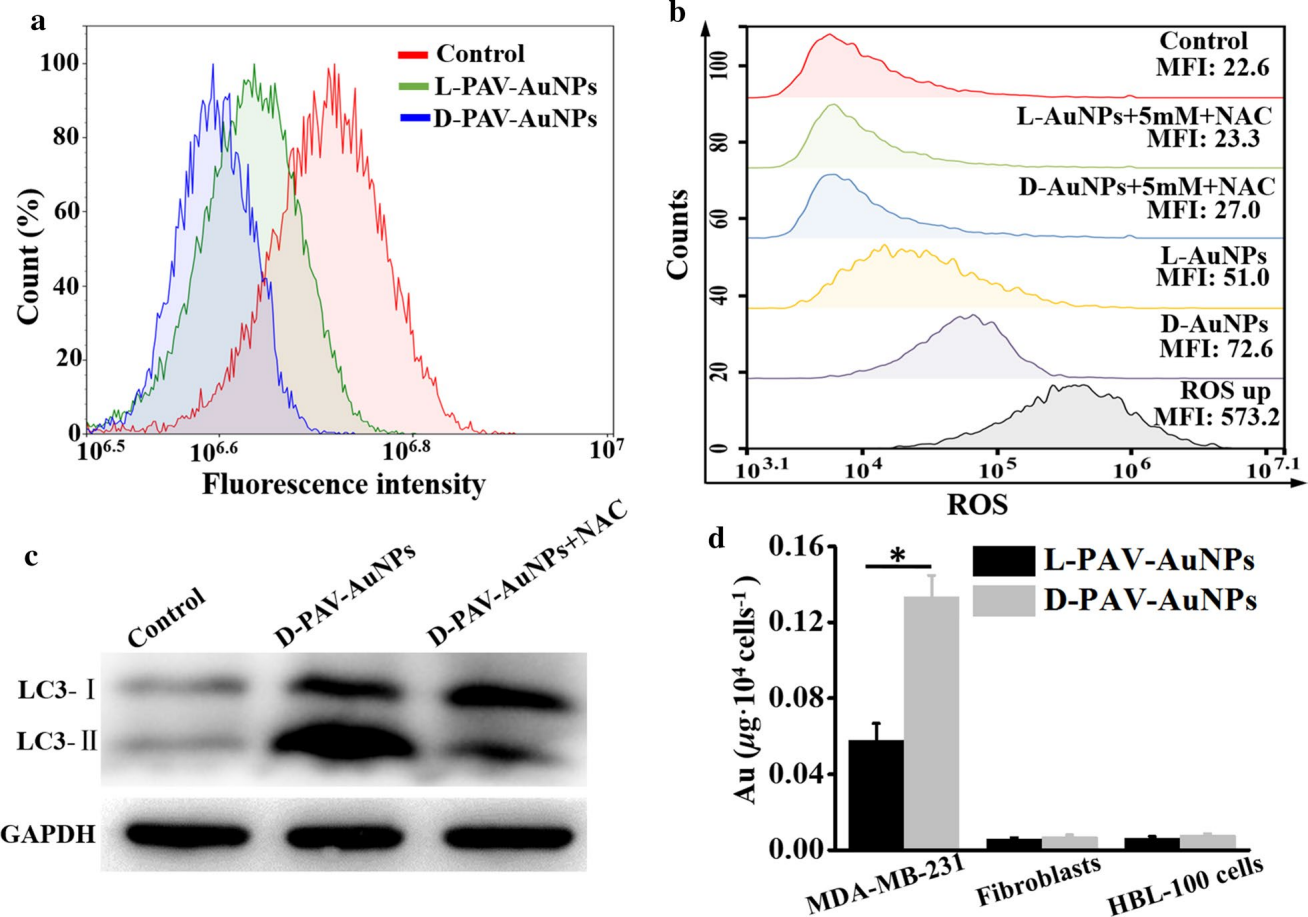
The possible chirality-dependent mechanisms of the PAV-AuNPs on autophagy-inducing activity. **a** l- and d-PAV-AuNPs reduced lysosome stability in MDA-MB-231 cells. **b** Generation of ROS in MDA-MB-231 cells exposed to l- or d-PAV-AuNPs with an Au concentration of 100 µg/mL in the presence or absence of antioxidants (NAC), respectively. ROS up is regarded as the positive control. The mean fluorescence intensity (MFI) is quantified using FCM. **c** The level of LC3-II in MDA-MB-231 and treated without (Control) or with d-PAV-AuNPs in the presence or absence of NAC for 24 h is determined by western-blotting, GAPDH served as loading control. **d** Cellular uptake of l(d)-PAV-AuNPs. Quantification of the Au amount per 104 cells is presented as the mean ± standard deviation (n = 4). * and ** present p < 0.05 and p < 0.01, respectively

To further verify whether the appearance of autophagy was associated with NP-induced cytotoxicity in MDA-MB-231 cells, quantitative study of cell viability after NP and autophagy inhibitor of 3-methyladenine (3MA) treatment was carried out. Firstly, the autophagy inhibitor of 3MA itself was observed to have no influence on the viability of the MDA-MB-231 cells (Fig. 3c). However, 3MA pretreatment significantly rescued cells (*p *< 0.05) from NP-induced cell death, particularly in cells treated with d-PAV-AuNPs (*p *< 0.01) (Fig. 3c). In view of the above results, we concluded that autophagic process is the mainly pathway of PAV-AuNPs induced MDA-MB-231 cells death. More importantly, the activation of autophagy was chirality-dependent, with d-PAV-AuNPs inducing more dramatic autophagy. It is known that lysosome plays an important role in autophagy and is considered to be the key organelles involved in autophagic cell death [43]. To determine whether the lysosome function in MDA-MB-231 cells could be interfered by PAV-AuNPs, the integrity of liposomal membranes was evaluated by using the FCM to examine the release of acridine orange (AO) from lysosomes into the cytosol [44]. Relative to the untreated cells, the intensity of the AO fluorescence signal was decreased in cells treated with PAV-AuNPs, regardless of surface chirality. The level of loss of the AO fluorescence intensity was greater in cells treated with d-PAV-AuNPs than in those treated with l-PAV-AuNPs (Fig. 4a). The stability of lysosomal membranes in the cells treated with PAV-AuNPs was coinciding well with the induction of autophagy.

Oxidative stress is believed to be one of the most important mechanisms of nanotoxicity, and the levels of intracellular ROS serve as reliable indicators of oxidative stress, which is essential for the induction of autophagy [45]. Therefore, the ROS levels in MDA-MB-231 cells treated with PAV-AuNPs were evaluated. Compared with the untreated control, there is a remarkable increase of ROS generation in MDA-MB-231 cells treated with PAV-AuNPs. Moreover, the level of ROS generation was higher in cells treated with d-PAV-AuNPs than in ones treated with l-PAV-AuNPs (Fig. 4b). It was observed that the interaction of NPs with cell surface receptors could result in activating of intracellular signaling cascades that induce formation of ROS [46]. Hence, we speculated that the surface chirality-variant ROS generation was largely due to the chirality-dependent cellular uptake as well as the specific interaction between receptors and biomolecules in cells and chiral molecules. It was known that ROS could be attenuated by pretreatment with antioxidant agents such as *N*-acetyl-cysteine (NAC). To further evaluate whether the autophagy was caused by the overproduction of ROS, the cancer cells were pretreated with 5 mM of NAC. As shown in Fig. 4b, the level of ROS in the cells pretreated with NAC was largely attenuated. Subsequently, the degree of transforming of LC3 I to LC3 II was examined, which also be reduced by the pretreatment of NAC (Fig. 4c). Overall, these results above strongly supported that the autophagy induced by PAV-AuNPs in cancer cells was largely attributed to the ROS overproduction.

Considering the differential biological effects of NPs could also be linked to their differential cellular uptake [13, 24, 47], quantitative measurements of internalized PAV-AuNPs were carried out by examining the element of Au content using the ICP-MS. The internalized amount of d-PAV-AuNPs was significantly larger than that of l-PAV-AuNPs in MDA-MB-231 cells (Fig. 4d), which is correlated well with our previous study that used the HepG2 cells due to the chirality-dependent interaction with the feature of cell membrane [17]. In contrast, internalized amount of PAV-AuNPs in 3T3 fibroblasts and HBL-100 cells was significantly smaller than that of PAV-AuNPs in MDA-MB-231 cells, and exhibited chirality-independent cellular uptake (Fig. 4d). Our previous work also reported that the level of ROS produced was associated with the amount of cellular uptake in lung adenocarcinoma A549 cells [23]. Therefore, the observed differential effects of surface chirality at the nanoscale on the autophagy of cancer cells, and the selectivity autophagy activation between cancer cells and normal cells are dependent to some extent, if not entirely, on the differential cellular uptake.

**Fig. 5:**
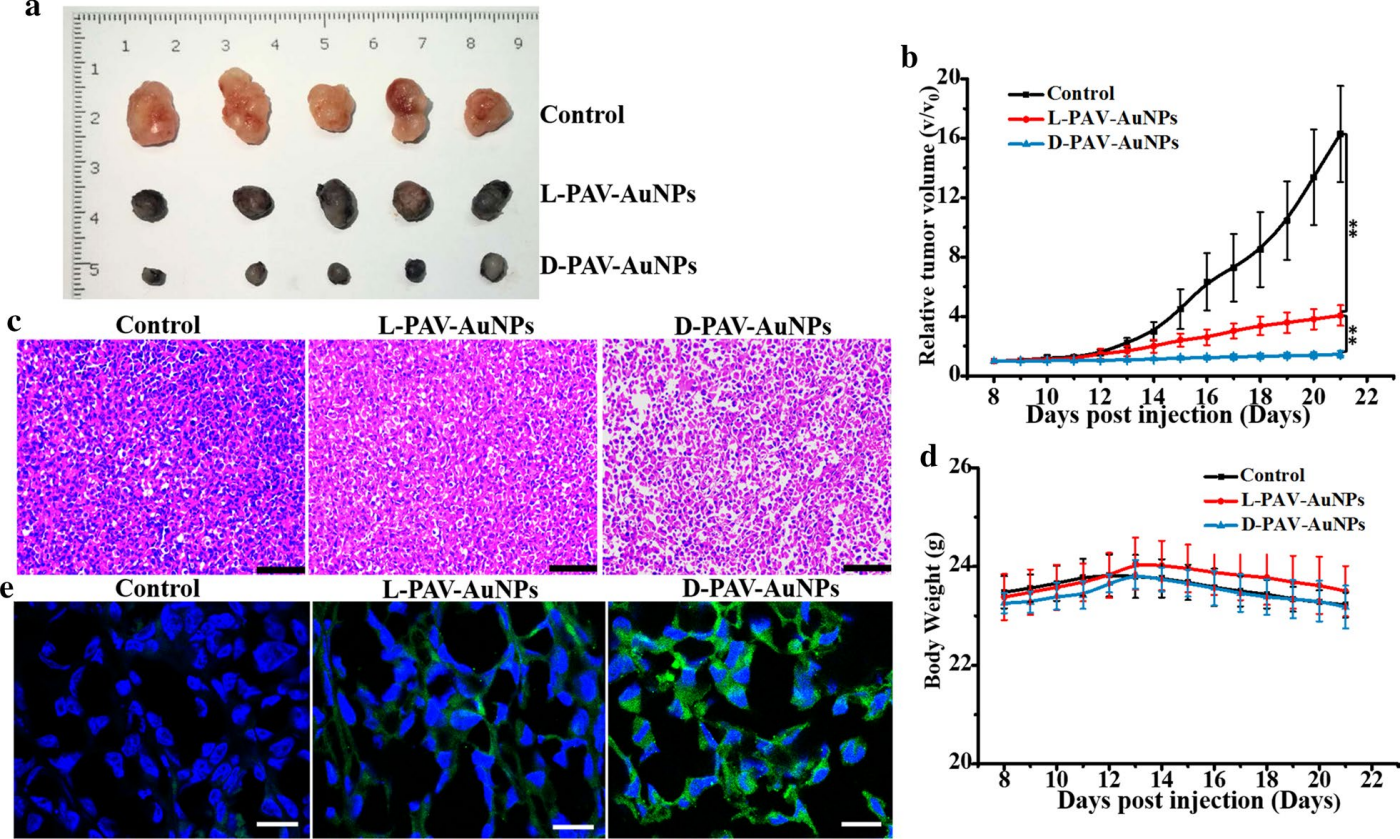
Antitumor efficacy on the tumor-bearing nude mice model. **a** Tumor tissues. **b** Relative tumor growth curve. **c** Pathological features of tumor tissues in nude mice treated with physiological saline or PAV-AuNPs. Tumor issues were H&E stained. **d** Changes in body weight during the course of treatment. **e** Immunohistochemical representative images for cryosections of MDA-MB-231 tumor tissues after intratumoral injection with physiological saline, l- and d-PAV-AuNPs, respectively. Stained with LC3-II; the entire nucleus was stained with DAPI. * and ** present p < 0.05 and p < 0.01, respectively

Recently, researchers have attached the importance to modulate autophagy and impede further tumor progress [9, 37, 48], particularly in autophagy-deficient cancers including breast, ovarian and prostate cancers. Inspired by the results above that chirality at the nanoscale could induce autophagic cancer cell death significantly while exhibit inactivation of autophagy for normal cells in vitro, we hypothesized that the employing of the chirality at the nanoscale to manipulate autophagy may provide a new antitumor solution for breast cancer therapy. Thus, the effect of PAV-AuNPs on tumor suppression in vivo was achieved by utilizing of the MDA-MB-231-bearing nude mouse. The mice were intratumorally injected with l- or d-PAV-AuNPs (50 μL of 1 mg/mL) every other day, and treated with physiological saline as control. Then, the tumor size, body weight of the mice, and pathological morphology of tumor tissues were evaluated. The process of tumor growth was observed and recorded for 14 days. As shown in Fig. 5a, b, the relative tumor volume of the control group increased more than that of the PAV-AuNPs treated group, regardless of surface chirality. Additionally, the growth inhibition of the tumor treated with d-PAV-AuNPs was stronger than the one with l-PAV-AuNPs treated. We further investigated the pathological morphology of all the tumor tissues. Compared to the control group, the PAV-AuNPs presented obvious breast tumor growth reduction and invasion inhibition, irrespective of the surface chirality. More importantly, d-PAV-AuNPs exhibited greater tumor growth reduction capability than l-PAV-AuNPs (Fig. 5c). Furthermore, body weight, as an indicator for the toxicity effects, was determined. Relative to the untreated control, none of the mice treated with l-PAV-AuNPs or d-PAV-AuNPs had significant variations in body weight, which inferred that there was no obvious toxicity with these treatments by intratumor injection in vivo (Fig. 5d).

To determine whether PAV-AuNPs could induce autophagy in vivo, an immunohistochemical method was used to detect the expression of LC3-II in tumor tissues [27]. Compared with the untreated control group, there was stronger immunofluorescence of LC3-II in the PAV-AuNPs treated group. In addition, the d-PAV-AuNPs treated group presented more immunofluorescence of LC3-II than l-PAV-AuNPs treated one (Fig. 5e). These results suggested that the in vivo activation of autophagy is chirality-dependent, with d-PAV-AuNPs are more effectively inducing autophagy in vivo. Overall, PAV-AuNPs with different chiral forms have shown the possibility of autophagy induction, which can be used as an effective regulator for tumor eradication in vivo.

**Fig. 6:**
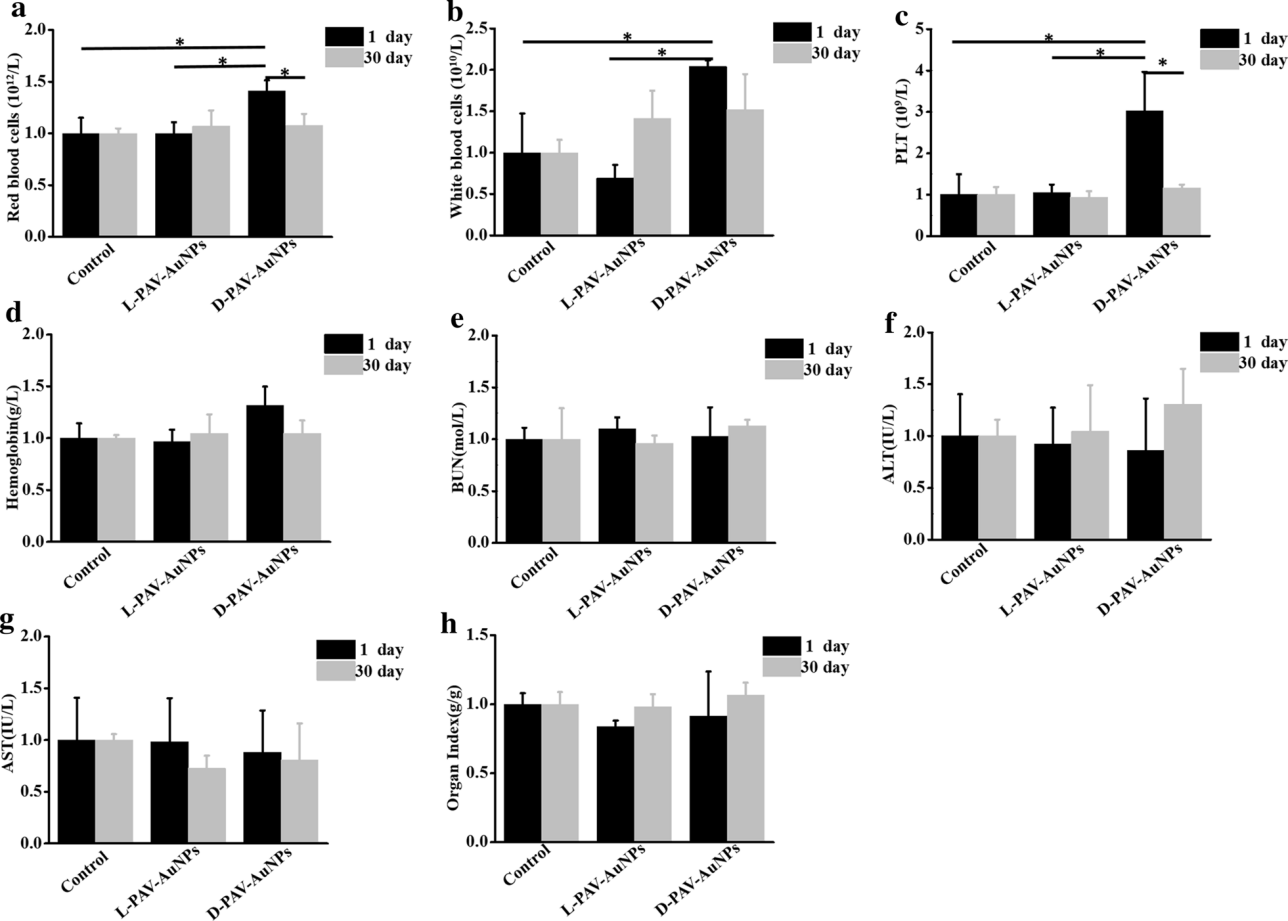
The blood chemistry analysis after injection of PAV-AuNPs in vivo. **a** Blood analysis of healthy Balb/c mice intravenous injected with PBS (PH = 7.4), l-PAV-AuNPs or d-PAV-AuNPs sacrificed at day(s) 1 and 30, including **a** red blood cells, **b** white blood cells, **c** platelet(PLT), **d** hemoglobin, **e** blood urea nitrogen (BUN), **f** alanine aminotransferase (ALT), **g** aspartate aminotransferase (AST), **h** organ index

**Fig. 7:**
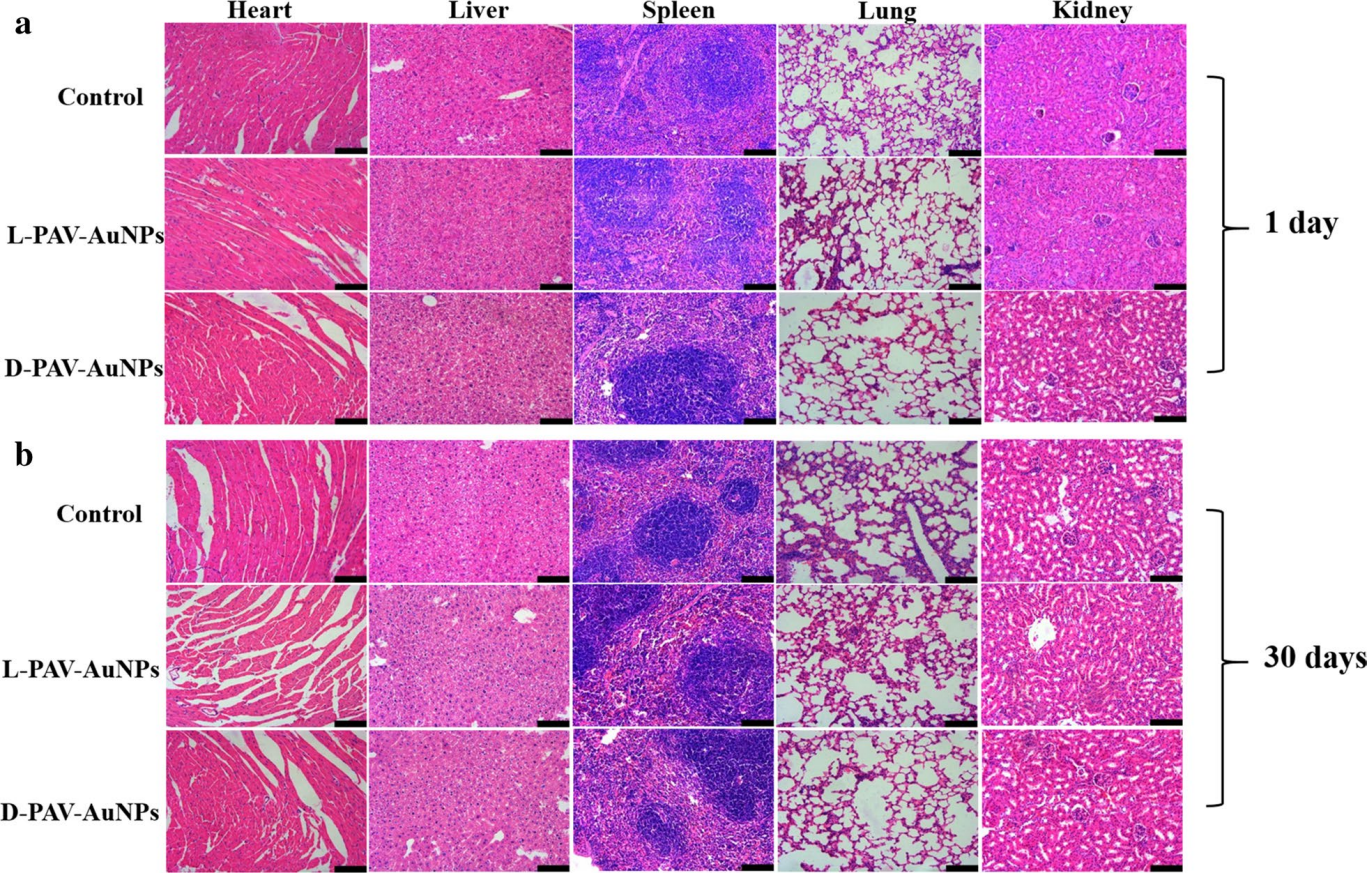
*In vivo* toxicity of PAV-AuNPs evaluation. H&E staining in main organs (liver, kidneys, spleen, heart, and lung) of the mice sacrificed at 1 (**a**) or 30 days (**b**) after intravenous injection of PBS or PAV-AuNPs. Scale bar stands for 100 μm

Based on above invigorated effect of tumor suppression in vivo, before harnessed to a novel therapeutic approach for clinical work, PAV-AuNPs has become obviously critical to further probe the distribution and toxicity of PAV-AuNPs in vivo. Firstly, the in vivo distribution of PAV-AuNPs was studied by determining the Au content in the major organs (heart, liver, spleen, lung and kidney) of health BALB/C mice after intravenous injection of PAV-AuNPs (1 mg/mL). In the early stages (1 day after injection), the PAV-AuNPs were mainly accumulated in the reticuloendothelial system (RES) organs such a liver and spleen, as expected the strong phagocytosis in RES organs (Additional file 1: Figure S3). However, the levels of PAV-AuNPs decreased largely in all the tested organs on 30 days after injection, indicating that PAV-AuNPs can be effectively cleared from mice with the time increasing (Additional file 1: Figure S3). Furthermore, the blood chemistry analysis after injection of PAV-AuNPs in vivo was also studied. After being injected intravenously at a certain time point (1 or 30 days), the blood chemistry analysis showed that only d-PAV-AuNPs caused significantly (*p *< 0.05) higher level of red blood cells, white blood cells (WBC), and blood platelet (PLT) (Fig. 6a–c) for 1 day, which may attribute to a certain level of acute inflammation, hematopoietic disorders or hematological system injury. Nevertheless, after being injected for 30 days, compared with control group, all results of the blood chemistry analysis of l- and d-PAV-AuNPs were in normal (*p *> 0.05) (Fig. 6e–h). Additionally, the major organs including the heart, liver, spleen, lung, and kidney were collected and stained with H&E for histology analysis after 1 or 30 days of intravenous injection. As presented in Fig. 7, there was no obvious tissue damage or inflammatory lesion in all major organs as well as no evident signs of abnormal mouse behavior. These results suggested that PAV-AuNPs have negligible toxicity in vivo for applications in biological medicine.

## Discussions

**Fig. 8:**
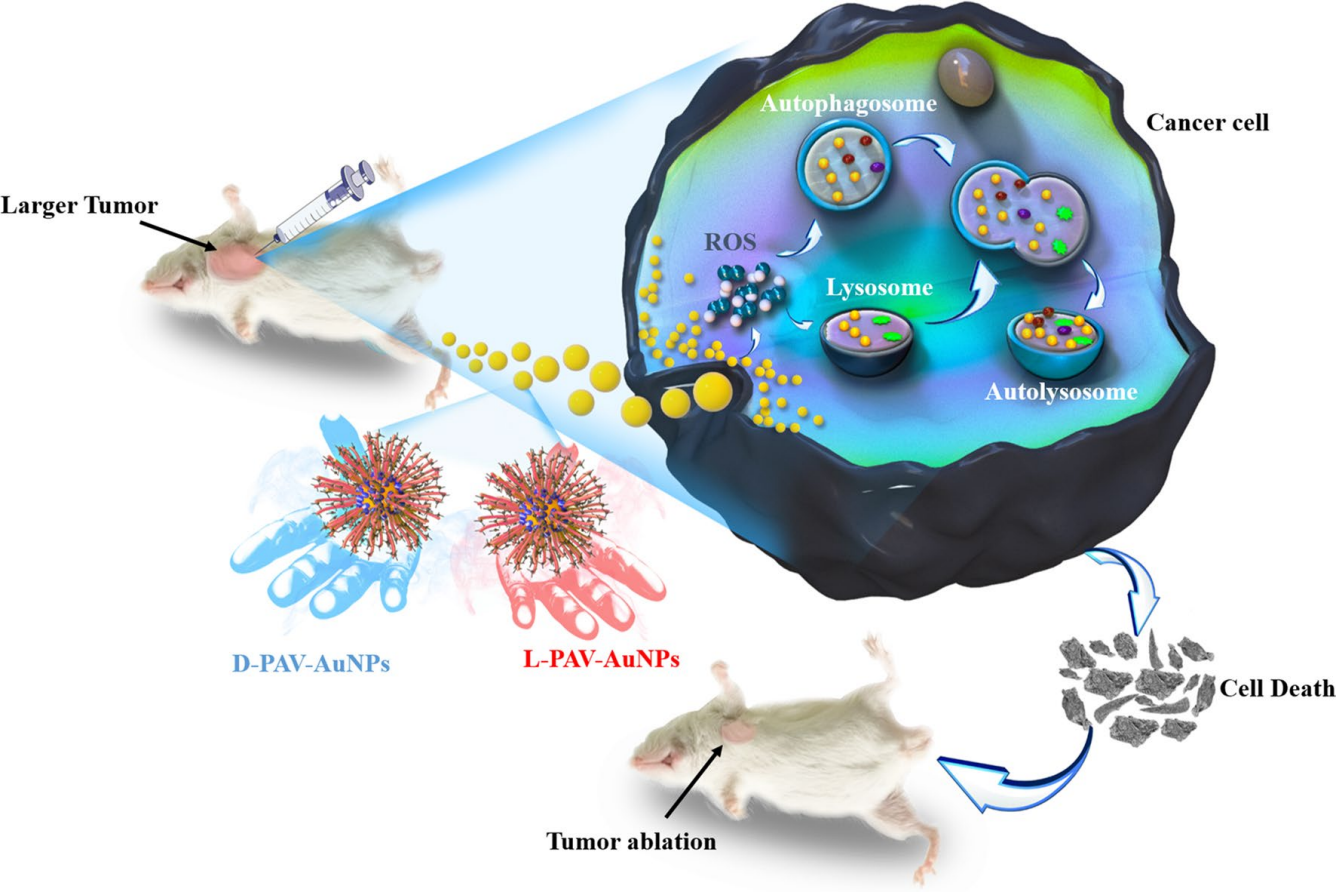
Schematic diagram of the mechanism of the chirality-dependent activation autophagy and their application in tumor therapy

We have developed a novel anticancer depots and evaluated their efficacy to eradicate tumors, triple-negative breast cancer in a murine model, as schematically summarized in Fig. 8. Our anticancer depots are equipped with a shell composed of chiral valine monomer units in PAV molecules that makes the nanoparticle target the cancer cells and avoid them interact with the host cells (normal cells) around the tumor sites. Moreover, the anticancer effects showed chirality-dependent and the d-PAV-AuNPs presented stronger anticancer effects, which was largely due to the chirality-dependent autophagy rather than the apoptosis. Take in together, these unique features allow our anticancer depots to completely ablate the TNBCs.

The central point of our work is that the chiral PAV-AuNPs could distinguish the normal cells and cancer cells, and show a chirality-dependent anticancer effects. Chirality, as one of the most distinctive biochemical signatures of life, has great influence on many biological events, for instance, the maintenance of normal functions of living cells [49, 50]. Pioneering works have revealed that cells can sense surface chiral signals and show differential interactions with enantiomorphous surfaces [18, 51]. Among these chiral molecules, amino acids have been widely used for studying the interaction between cells and chiral surface due to their versatility and biocompatibility [51]. For example, Gammon et al. investigated sequence-specific cell uptake characteristics of Tat basic domain and related permeation peptides with an emphasis on residue chirality, length, and modified side chains [52]. It was observed that the length, sequence and types of chelation domain impacted peptide uptake into cells. More importantly, with all the other factors are same, once the chirality of the peptide sequence was changed from l to d, uptake values increased up to 13-fold. Furthermore, the eight essential amino acids showed stronger chirality-dependent cell uptake effect and would appear to optimize the permeation sequence for both Tat basic domain and poly-Arg peptides [52]. Valine is one of the eight essential amino acids for the human body, and plays essential roles in a wide variety of physiological processes [53–55]. Sun et al. reported stereo selective cell behaviors on a pair of chiral brush films, which were composed of a chiral unit of acryloyl derivatives of l(d)-valine (AV) [33]. A fibroblast-like cell line—COS-7 cells were cultured on l(d)-valine based films (l(d)-PAV). It was found that the adhesion, spreading, growth and assembly processes of cells were significantly different on two films. The cells preferred to connect to each other and spread on the l-PAV film as interlinked clusters, whereas those on the d-PAV film tended to remain isolated stacks with lower spreading extent [33]. More recently, they further studied the influence of the molecular structure of the chiral units on this chiral effect by substituting the l(d)-Val units with other two kinds of aliphatic amino acids, l(d)-alanine (Ala), and l(d)-leucine (Leu) [51]. The only difference among these three amino acids is the size of the hydrophobic side groups. It was observed that the smallest Ala units led to the weakest chiral effect in which differential cell behaviors with statistical significance could only be observed after long periods of cell incubation. However, for polymer films based on the Leu units, a more distinct chiral effect was found compared with those based on Val units (as reported above) [51]. The above results revealed that the amino type could influence the chiral effects on biological systems. Our previous works also prepared chiral surface based on the valine and studied stereoselective interactions between cells and chiral interface materials [13, 20, 23, 56]. We observed that cancer cells (e.g., A549 cells and HepG2 cells) prefer to internalize the d-PAV-AuNPs through the possible preferable interaction between the l-phospholipid-based cell membrane and the d-enantiomers [42], while the bone marrow mesenchymal stem cells uptake more l-PAV-AuNPs than the d-PAV coated ones [20]. Such effects were attributed to the different cell types. More interestingly, the PAV-AuNPs could discriminate model tumor cells (MDA-MB-231 cells) from normal cells (3T3 fibroblasts and HBL-100 cells). Upon contact with the complex biological systems, the proteins will be progressively and selectively adsorbed on the NPs surface unless they have been designed to do otherwise, which was defined as “protein corona” [55, 57–59]. Increasing evidence suggest that the protein corona define the biological interactions of NPs [60, 61]. Thus, we make assumptions that the protein corona in PAV-AuNPs could selectively interact with cancer cells from normal cells and exhibit chirality-selective. For example, transferrin receptor is an ubiquitous human cell surface glycoprotein related to cell proliferation [62], and it is expressed more abundantly in malignant tissues than in normal tissues [63]. The affinity of transferrin to the surface of cancer cells is 10–100 times greater than that of normal cells [64]. Furthermore, the abundance of transferrin receptor in malignant tissues of human breast has been demonstrated [65]. Li et al. reported that the transferrin–transferrin receptor-mediated cellular uptake of gold nanoparticles is six times of that in the absence of this interaction [66]. More recently, Wang et al. showed that l(d)-penicillamine coated gold nanoparticles could strongly interact with the transferrin and realize the tumor targeting [67]. For d-penicillamine modified AuNPs, the binding transferrin-related residues are positioned outward, which facilitates the interaction between transferrin and transferrin-receptor, while for the l-penicillamine coated AuNPs, they face inward and hinder the interaction. Meanwhile, transferrin undergoes different degrees of changes in its secondary structures after its interaction with different chiral surfaces of AuNPs [67]. Moreover, our previous work also observed that the albumin showed secondary structures change after being adsorbed on l(d)-PAV-AuNPs [22]. As expected, some proteins from serum such as transferrin adsorbed on PAV-AuNPs could strongly and selectively interact with breast cancer cells. Moreover, the proteins adsorbed on d-PAV-AuNPs had a stronger binding to cancer cell receptors than on l-PAV-AuNPs. The chiral molecule interact with other biomolecules (e.g., receptor or proteins) via hydrogen bonding (H-bonding), hydrophobic interaction, electrostatic attraction, and dipole–dipole interaction, etc. [68]. Thus, the molecular functional groups, charges and hydrophobic units in chiral molecules may affect their chiral effects.

In addition, another insight was that NP could induce cancer cellular injury through autophagy pathway, which exhibited chirality-dependent. The possible mechanism of the chirality-dependent activation autophagy in cancer cells was attributed to the larger intracellular accumulation of the d-PAV-AuNPs, which leads to higher ROS production. According to our results (Fig. 3c), we speculate that l(d)-PAV-AuNPs induced cancer cell autophagy may occur through the phosphatidylinositol 3-kinase (PI3K) signaling pathway (an important pathway in autophagy regulation) [68], because it could be efficiently inhibited by 3-MA, which is considered to be a specific inhibitor of PI3K activity. Taken together, it is probable that the ROS induced by the l(d)-PAV-AuNPs can trigger autophagy both directly and indirectly via inhibition of the classical autophagy signaling pathway, phosphatidylinositol 3-kinase/protein kinase B/mammalian target of rapamycin (PI3K/Akt/mTOR).

Finally, our anticancer depot especially the d-PAV-AuNPs could be used as an efficient anticancer agent for future clinic application, which was demonstrated by the largely tumor suppression and negligible toxicity in vitro and in vivo.

## Conclusion

In summary, this work provides new insights into NP induced cancer cellular injury by demonstrating differential autophagy associated with the chirality of PAV coating. The cytotoxicity induced by PAV-AuNPs in model breast cancer cells (MDA-MB-231 cells) was mainly through autophagy pathway, which was chirality-dependent, with d-PAV-AuNPs exhibiting efficient autophagy-inducing activity in vitro and in vivo. In contrast, the PAV-AuNPs presented autophagy inactivation in the model normal cells (3T3 fibroblasts and HBL-100 cells), regardless of the surface chirality. Compared with l-PAV-AuNPs, d-PAV-AuNPs exhibited excellent internalization, a crucial first step in obtaining the intracellular accumulation of d-PAV-AuNPs. The larger intracellular accumulation of the d-PAV-AuNPs can lead to higher ROS production and more serious lysosome function interfered, which dramatically, if not entirely, gave rise to the occurrence of chirality-dependent autophagy. Additionally, what’s exciting is that stereospecific d-PAV-AuNPs could act as a high efficient artificial autophagy-inducing agent for tumor autophagy induction, which was demonstrated by the largely tumor suppression and negligible toxicity in vitro and in vivo. Identification of this chirality-dependent autophagy of NPs provides an important insight that chiral effect can act as a novel strategy for designing bio-interface materials for cancer cell therapy especially TNBC.

## Additional file

**Additional file 1: Figure S1.** Stability in different solutions as indicated of l-PAV-AuNPs and d-PAV-AuNPs. (a) Photos and (b) UV–Vis-NIR of l-PAV-AuNPs or d-PAV-AuNPs in different solutions including saline, PBS, cell medium, fetal bovine serum and dilution of whole blood of the mice for 3 days. **Figure S2.** The toxicity study of l/d-PAV-AuNPs. (a) Dose- and chirality-dependent cytotoxicity of l/d-PAV-AuNPs in MDA-MB-231 cells, 3T3 fibroblasts and HBL-100 cells respectively. (b) Apoptosis rates of the MDA-MB-231 cells, 3T3 fibroblasts and HBL-100 cells treated with PAV-AuNPs, respectively. FCM analysis was tested via Annexin V-FITC and PI as probes. (c) Expression levels of LC3 in MDA-MB-231 cells, 3T3 fibroblasts and HBL-100 cells with PAV-AuNPs treatment, separately. GAPDH was used as a loading control. **Figure S3.** Biodistribution of PAV-AuNPs in vivo. The in vivo biodistribution of PAV-AuNPs was analyzed by testing the Au content in main organs (liver, kidneys, spleen, heart, and lung) of mice at 1 and 30 days post intravenous injection, separately. * and ** present *p *< 0.05 and *p* < 0.01, respectively.

## Abbreviations

TNBC: triple-negative breast cancer
CCK8: cell counting Kit-8
3-MA: 3-methyladenine
TEM: transmission electron microscopy
DLS: dynamic light scattering
ROS: reactive oxygen species
AO: acridine orange
NAC: *N*-acetyl-cysteine
WBC: white blood cells
PLT: platelet
