# Supplementary material for: Chiral polymer modified nanoparticles selectively induce autophagy of cancer cells for tumor ablation
Source: J Nanobiotechnology. 2018 Jul 11;16:55. doi: 10.1186/s12951-018-0383-9 (PMC6040058; doi:10.1186/s12951-018-0383-9)
Supplement: Supplementary file 1 — Additional file 1: Figure S1. Stability in different solutions as indicated of l-PAV-AuNPs and d-PAV-AuNPs. (a) Photos and (b) UV–Vis-NIR of l-PAV-AuNPs or d-PAV-AuNPs in different solutions including saline, PBS, cell medium, fetal bovine serum and dilution of whole blood of the mice for 3 days. Figure S2. The toxicity study of l/d-PAV-AuNPs. (a) Dose- and chirality-dependent cytotoxicity of l/d-PAV-AuNPs in MDA-MB-231 cells, 3T3 fibroblasts and HBL-100 cells respectively. (b) Apoptosis rates of the MDA-MB-231 cells, 3T3 fibroblasts and HBL-100 cells treated with PAV-AuNPs, respectively. FCM analysis was tested via Annexin V-FITC and PI as probes. (c) Expression levels of LC3 in MDA-MB-231 cells, 3T3 fibroblasts and HBL-100 cells with PAV-AuNPs treatment, separately. GAPDH was used as a loading control. Figure S3. Biodistribution of PAV-AuNPs in vivo. The in vivo biodistribution of PAV-AuNPs was analyzed by testing the Au content in main organs (liver, kidneys, spleen, heart, and lung) of mice at 1 and 30 days post intravenous injection, separately. * and ** present p < 0.05 and p < 0.01, respectively. [file 12951_2018_383_MOESM1_ESM.doc]

Additional information

**Chiral polymer modified nanoparticles selectively induce autophagy of cancer cells for tumor ablation**

Long Yuan^1#^, Fan Zhang^1#^, Xiaowei Qi^1^, Yongjun Yang^2^, Chang Yan^3^, Jun Jiang^1^*, Jun Deng^4^*

1. Department of Breast Surgery, Southwest Hospital, Third Military Medical University (Army Medical University), Chongqing 400038, China.
2. Medical Research Center, Southwest Hospital, Third Military Medical University (Army Medical University), Chongqing 400038, China.
3. Department of Cardiology, Southwest Hospital, Third Military Medical University (Army Medical University), Chongqing 400038, China.
4. Institute of Burn Research, Southwest Hospital, State Key Lab of Trauma, Burn and Combined Injury, Third Military Medical University (Army Medical University), Chongqing 400038, China.

^#^ These authors contributed equally to this work.

* Corresponding authors.

E-mail addresses:

Long Yuan: [ylong2002013479@163.com](mailto:ylong2002013479@163.com), Fan Zhang: zhangfan316@163.com,

Xiaowei Qi: qxw9908@foxmail.com, Yongjun Yang: 2005034145@163.com,

Chang Yan: yanc918@126.com, Jun Jiang: jcbd@medmail.com.cn,

Jun Deng: [djun.123@163.com](mailto:djun.123@163.com).


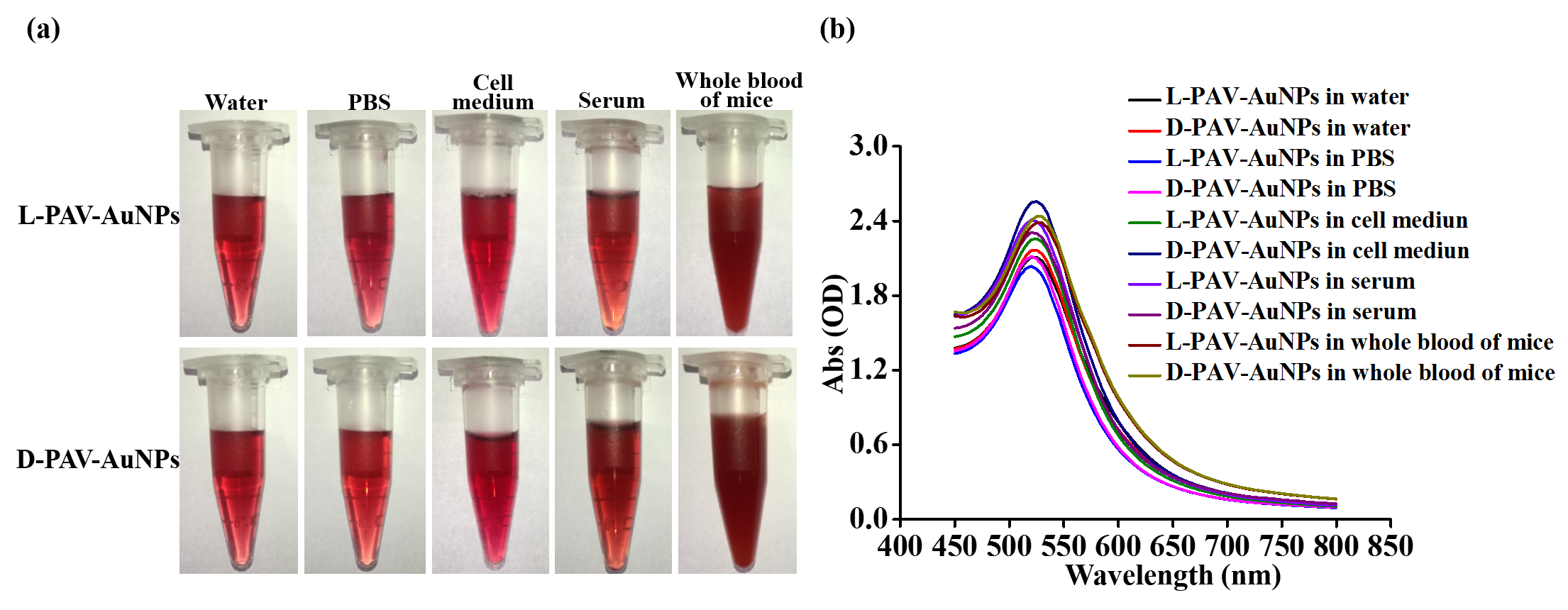


**Additional file 1: Figure S1.** Stability in different solutions as indicated of L-PAV-AuNPs and D-PAV-AuNPs. (a) Photos and (b) UV-vis-NIR of L-PAV-AuNPs or D-PAV-AuNPs in different solutions including saline, PBS, cell medium, fetal bovine serum and dilution of whole blood of the mice for 3 days.


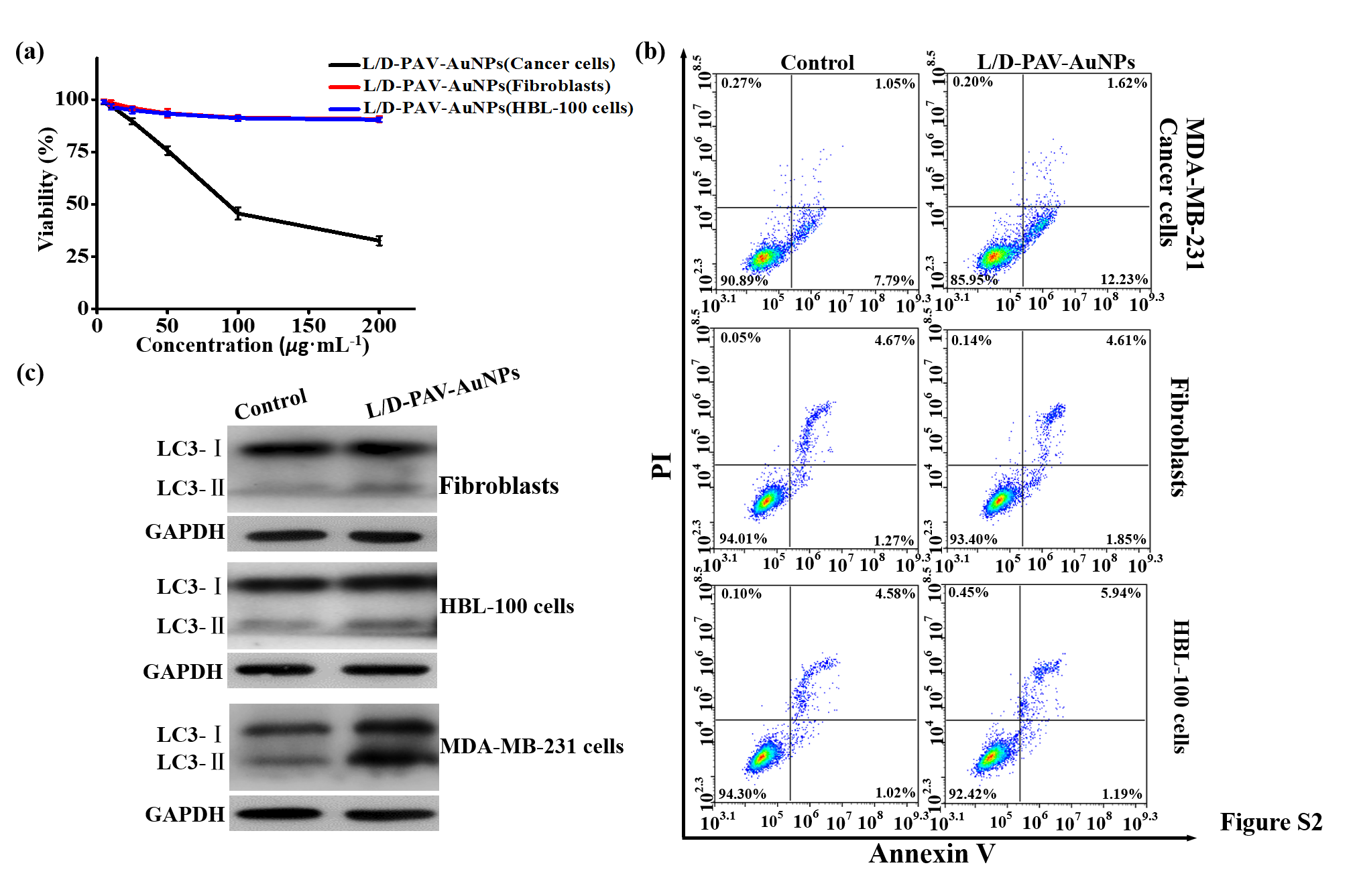


**Additional file 1: Figure S2.** The toxicity study of L/D-PAV-AuNPs. (a) Dose- and chirality-dependent cytotoxicity of L/D-PAV-AuNPs in MDA-MB-231 cells, 3T3 fibroblasts and HBL-100 cells respectively. (b) Apoptosis rates of the MDA-MB-231 cells, 3T3 fibroblasts and HBL-100 cells treated with PAV-AuNPs, respectively. FCM analysis was tested via Annexin V-FITC and PI as probes. (c) Expression levels of LC3 in MDA-MB-231 cells, 3T3 fibroblasts and HBL-100 cells with PAV-AuNPs treatment, separately. GAPDH was used as a loading control.


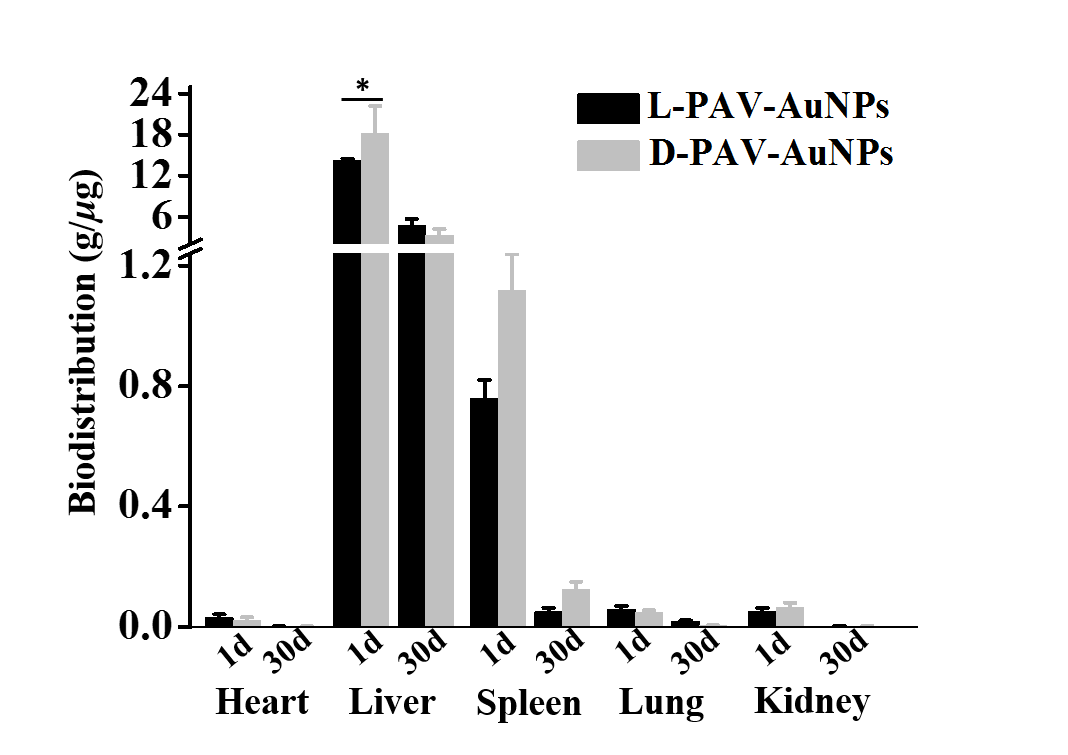


**Additional file 1: Figure S3.** Biodistribution of PAV-AuNPs *in vivo.* The *in vivo* biodistribution of PAV-AuNPs was analyzed by testing the Au content in main organs (liver, kidneys, spleen, heart, and lung) of mice at 1 and 30 days post intravenous injection, separately. * and ** present *p* < 0.05 and *p* < 0.01, respectively.
